# Supplementary material for: What Strategies Do Healthcare Providers Use to Promote Adolescents’ Vaping Cessation? A Scoping Review
Source: Int J Environ Res Public Health. 2025 May 27;22(6):839. doi: 10.3390/ijerph22060839 (PMC12193372; doi:10.3390/ijerph22060839)
Supplement: Supplementary file 1 [file ijerph-22-00839-s001.zip › ijerph-3539058-supplementary.pdf]

Supplementary Table S1 : Key findings from the included studies

First Author (Year)	Knowledge	Attitude	Practice	Training	Limitations
Brown-Johnson et al. (2016)	1.27% Providers knowledge on the general safety and addictiveness of-cigarette	1.47% of providers' responses represented a negative attitude toward e-cigarettes.<br><br>2.20% were positive; positive attitudes toward e-cigarettes may have been more highly approved by patients than responses coded as negative or neutral.	Half or more of the responses were coded as a negative attitude toward e-cigarettes for the remaining answer themes of side effects and harms, nicotine health risks, use in the presence of medications or pre-existing conditions, the extent of research evidence, nicotine health risks, and level of product regulation (data not shown for themes occurring in less than 10% of responses).	Only 5% had received training on addressing their practice	1.The providers were identified, and their responses were posted publicly, which may have constrained their answers,<br><br>2.Study did not assess patient e-cigarette experience or information sources guiding provider opinions
Chin et al. (2022)	1.Lack of knowledge for e-cigarette use (47.6%) among the providers.<br><br>2.Among all respondents, only (6%) completed education on e-cigarettes or tobacco counselling in the form of online modules, conferences, grand rounds presentations, community discussions, or through research updates	1.80% of respondents agreed or strongly agreed that e-cigarettes contain nicotine, is a serious problem among youth.<br><br>2.70% agreed or strongly agreed that e-cigarettes use increases the risk of traditional tobacco use.<br><br>3.30% of providers reported that discussing tobacco would not produce behavioural changes	1 During the screening, 98% asked about tobacco use, while 18% never asked about e-cigarettes use<br>2. Discussion of harms and assistance in cessation for e-cigarettes use was reported in a lower percentage of providers (58% r ($p < 0.0001$) whereas 80% respondent felt confident about discussing the harms of tobacco.<br>3. Less than a quarter of respondents felt confident in counselling regarding nicotine cessation using supports such as quit lines or online resources. Only 2% of respondents felt confident in prescribing NRT.<br>4. Providers were more likely to assist in the cessation of tobacco use compared to e-cigarettes use ($p <$<br>0.05).<br>5. Providers reported insufficient time within clinic visits (80%) for e-cigarettes and tobacco.	-	1.Survey data designed to collect information from the respondent reported asking tobacco and e-cigarette, so data was not exclusively on e-cigarette.

Cano Rodriguez et al. (2021)	-	-	During four months of the intervention screening for ENDS use started at two patients (0.48%) at baseline in 1st intervention and progressively.<br>increased after the 2nd intervention to 243 patients (62%), 320 patients (80%) in 3rd, and 456 (90%) by the end of the study.<br>First intervention: educating the adolescent staff on the importance of direct screening for ENDS use and the use of a paper screening questionnaire changes in screening rate to 17% (The addition of a specific question for ENDS uses in electronic health records (software for the patient record) resulted in a screening rate of 95%		1.Small sample size of healthcare providers.<br>2.Conveniencesampling, participants may not represent the health care providers as a whole.
Gorzkowski et al. (2016)	All participants reported some general knowledge about e-cigarettes,<br>1. (81%) Knew that most e-cigarettes contained nicotine,<br>2. (51%) Described them as addictive<br>3. (43%) Had knowledge that they came in youth-friendly flavours<br>4. (84%) Had some knowledge on e-cigarette laws<br>5.(76%) paediatricians were unsure about the effect of second-hand e-	1. Participants likely to believe-cigarettes are safer than cigarettes (40%) were concerned about the limitations of the evidence for e-cigarette harm<br>2. (92%) paediatricians felt uninformed about the health effects of e-cigarettes,<br>3. (76%) paediatricians were unsure about the effect of second-hand e-cigarette vapor or emissions<br>4.(22%) paediatricians expressed feeling wary of e-cigarettes and sceptical about their safety	1.Most (95%) paediatricians did not systematically screen for e-cigarette use<br>2.. (65%) of paediatricians had never discussed them35% stated it had happened at least once.<br>3.. Barriers to discussing e-cigarettes are competing priorities.<br>4. (8%) paediatricians reported that they had faced systemic difficulties when adding screening questions to their electronic health record.	-	1. Data collected from the paediatrician who attended the American academy of paediatrics',<br>2.This small qualitative study may not allow for the exploration of a full range of responses.

	cigarette vapor or emissions<br>6. Several respondents (40%) were concerned about the limitations of the evidence for e-cigarette harm				
Gorukanti et al.<br>(2022)	1. Perceived addictiveness of cigarettes versus e-cigarettes was (p<0.0001),<br>2. Perceived harmfulness for cigarette use compared with e-cigarette use was(p<0.0001)	1. Clinicians perceived prevalence of e-cigarette use was 10%<br>2. Clinicians very unlikely would recommend an adolescent switching to e-cigarette who smokes 2-3 cigarettes a day<br>3. Clinicians' perception of adolescents who use e-cigarettes were more likely to go on later and use cigarettes.	1. Clinicians reported very comfortable talking to their adolescent patients about e-cigarettes.<br>2. Clinicians were somewhat confident in discussing the health impact of e-cigarettes.<br>3. Clinicians screened 50% of their adolescent patients for e-cigarette use and 100% for cigarette use(p<0.0001).<br>4. Clinicians counselled 90% of adolescent patients for cigarette use and 20% for e-cigarette patients(p<0.0001).		1. Survey data designed to collect information from the respondent both from adolescents and young adults for different substance use, not exclusively on e-cigarettes.<br><br>2.During the data collection time, one of the substances (marijuana) was broadly legalised, which could influence the clinician's perspective and practices.
Metcalf et al.<br>(2022)	1. Self-identified vaping expert (primary care physicians) and nonexpert (non-primary physicians), both participants were fairly knowledgeable about several common myths about vaping (vaping is a good option for smoker, vaping tetrahydrocannabinol is the main problem and helping vaping patient is just like helping smoker)	Healthcare providers were interested in the among the checklist of 10 topics, percentage of participants interested in each case ranged from52%to 80%<br><br>1. non-experts were interested in the health effects of second- and third-hand vaping (87%)<br><br>2. Most endorsed topic chosen by the providers were pros and cons of vaping about 80% (20/25)	-	Healthcare providers agreed the need training among the selected 8 clinical skills<br><br>1. All of the Health care professionals (100%) agreed or strongly agreed with the need for Treatment recommendations for	1. Selection bias as participants were recruited from a list of health professionals who undertake their other substance use courses.<br><br>2.Convenience sampling, participants may not represent the health care providers as a whole.

	differences was not statistically significant<br>2. A larger differences (1.3 out of 5) between experts and nonexperts suggested that among myths was larger knowledge gap present about this topic.	3.non-experts also interested in health effects of second and third hand vaping (`n=13,87%)		vaping patient/ Patients counselling<br>2. Evaluation and treatment of the health effects of e-cigarette users (100% agreed or strongly agreed)<br>3. Experts agreed on the need of training on Talking with the parents about vaping prevention and helping their adolescent child to quit. (100% agreed or strongly agreed)<br>Expert rated the need of training higher than nonexperts (P=.01)	
McGee et al. (2021)	1.The majority (87.6%) agreed that they want to improve their knowledge by learning more about e-cigarettes.<br>2. Gaps in knowledge with the 5As + 5Rs model for tobacco counselling (66.7%)	-	1.Over half (55.6%) of providers reported confidence in their ability to talk with adolescents about e-cigarettes<br>2.About three quarters (74.0%) identified as they had barriers to counselling<br>3.Other barriers were not knowing where to refer (43.8%) and how to treat (27.8%) patients	-	1.Participants were providers practising in a single safety net institution limiting generalizability of findings.<br>2.Social desirability bias,

	• chemical content of e-cigarettes (55.4%),<br><br>• symptoms of EVALI (51.8%).<br><br>• Different types of e-cigarettes (47.9%)				
Oliver et al. (2022)	1.92% and 91% of the sample responding correctly about EVALI observed in pre-test; there is no significant difference in the post test	No significant changes were observed in these beliefs and attitudes at post-test.<br><br>1. E-cigarettes are safer than combustible tobacco<br><br>2. Using e-cigarettes may cause adolescents to initiate the use of tobacco 3. It is important to discuss e-cigarettes with adolescents, and parents of adolescents need to know about e-cigarettes.	A range of improvement was observed in screening methods and frequency from session 1:0% of the time to 6: 100%<br>•Improved comfort level in talking to a patient about e-cigarettes Mean SD 3.27(0.77) (P = .008)<br>•Counselling adolescent patients on nicotine replacement products Mean SD 2.91(0.61) (P = .046)<br>• Formal screening about vaping and tobacco was the least common practice (Mean=2.04 and mean = 2.07) while asking adolescent patients about tobacco/cigarette use was the most common (Mean = 4.00)	-	1.Retrospective analysis with evaluation data, many participants did not complete post-tests, even when incentivized.<br><br>2.Multiple attrition points<br><br>3.As the post-test was offered at a single time following all three sessions<br><br>4.Unmeasured variability exist in the time between exiting the program and completing the post-test.
Peterson et al. (2018)	Providers felt Conversational barriers because of lack of knowledge on<br>1. The degree of harm associated with e-cigarettes medical knowledge about e-cigarettes<br>2. Unfamiliarity with adolescents' motivations and slang,	Providers' expressed interest in the topics on the discussion were.<br>1. It is important to discuss both known and unknown risks of e-cigarettes vaping as a gateway to other forms of tobacco or substance use.	1. Strategies used to facilitate discussion with adolescent patients were<br>• Emphasizing medical uncertainty about the health effects of vaping<br>• Utilizing motivational interviewing, and<br>• Providing outside resources.<br>2. Reported of Inadequate screening tools<br>3. lack of time during the visit Allocated time is insufficient during clinic visit.	Providers emphasizing the lack of e-cigarette regulation for both products and advertising	1.Social desirability bias may have affected responses or prompted withholding of information.<br><br>2.Did not assessed knowledge and efficacy between provider types.

Pepper et al. (2015)	1.Most physicians (89%) wanted to learn more information about at least one e-cigarette topic<br>2.Popular topic for desired education was the potential health harms of use (75%), Potential health harms of second-hand aerosol (55%),4Whether using e-cigarettes leads to smoking (53%)	1.Physicians' primary concern about e-cigarettes was most often the potential health harms of use (50%), Using e-cigarettes may lead to smoking (35%)<br>2.breathing second-hand aerosol might be harmful (5%),	1.One-third of physicians (34%) reported having ever discussed e-cigarettes during an adolescent's visit<br>2. Initiation of the discussion by the physicians (46%), followed by the parent (30%), the adolescent (23%), or someone else (2%).<br>3. Physicians routinely screening adolescent patients for cigarette smoking compared few routinely toe-cigarette use (86% vs. 14%; p < .001).<br>4. Routine counselling was similarly more common for avoiding cigarette smoking than for avoiding e-cigarette use (79%vs. 18%; p < .001)<br>5.Family medicine physicians had higher odds of engaging in the prevention of e-cigarette use than (26% vs. 18%)<br>6. The minority of physicians would recommend e-cigarettes for smoking cessation, although endorsement varied by patients' age (36% would recommend to adults vs. 24% would recommend to adolescents' (< .001)<br>7. Physicians who agreed with the statement that e-cigarettes were less harmful than cigarettes were more likely to be willing to recommend them to adolescents for smoking cessation (35% vs. 17%; adjusted odds ratio (OR;2.63) .<br>8. Family medicine physicians had higher odds of engaging in prevention of e-cigarette use than (26% vs. 18%; adjusted OR1.57).	Support for regulation of e-cigarettes.<br>• Most agreed that e-cigarette use should be prohibited where smoking is (81%).<br>• laws for banning flavours for e-cigarettes (68%).<br>• All respondents also agreed that laws should prevent minors from buying e-cigarettes (91%)<br>• Advertisements targeting youth should be banned (86%).	1. Nurse practitioners or physician assistants who also provide primary care to adolescents were excluded from the study.<br><br>2.Did not ask about physicians' prior clinical experiences with e-cigarettes.<br><br>3.E-cigarettes images and presented at the beginning of the survey of the study better represented older models than newer model.
Pepper et al. (2014)	1. Nearly all providers (92%) had heard of e-cigarettes among them 83% reported that they knew "a little" or "nothing at all" about e-cigarettes	1. Moderately agreed that e-cigarettes are safer than regular cigarettes and smokeless tobacco (mean 2.7, SD .8).<br>2. providers expressed considerable concern that e-cigarettes could be a	1. More than one in ten respondents (11%) reported treating at least one adolescent patient who had used e-cigarettes<br>2. (53%) of who had heard of e-cigarettes were either "somewhat" or "very" uncomfortable talking to patients about them	-	1.Cross-sectional design and a low response rate, leads to nonresponse bias

	2. Family medicine physicians were more likely to be aware of e-cigarettes than either paediatricians or nurse practitioners (97% vs. 88%)	gateway to other tobacco use (mean 3.0, SD .8)<br>3. More than half of providers (53%) who had heard of e-cigarettes were either "somewhat" or "very" uncomfortable talking to patients	3. Provider's age was positively associated with comfort in discussing e-cigarettes with a patient (r=.09).<br>4. Providers who believed e-cigarettes were safer than other tobacco products, less likely to feel it was important to discuss e-cigarettes with patients (r =-.21) or parents of patients (r=-.18).		2. Not generalizable to other types of healthcare providers and to providers in other areas
Rajiv Singh et al. (2024)	1.There is mixed awareness among GPs about additional constituents such as propylene glycol, vegetable glycerine, THC, and flavourings, with 45% believing all of these are present.<br>2. Majority 94% believed e-cigarettes contain nicotine either always or sometimes.	1. About 45% of GPs believed 20-50% of 12–17-year-olds in Australia have tried e-cigarettes.<br>2. 98% agreed on health risks that e-cigarettes could cause respiratory distress.<br>3. Some GPs believed that non-smokers who use e-cigarettes are more likely to start smoking.<br>4. 62% Disagreed that e-cigarettes are less addictive than conventional cigarettes.	1. Only 34% GPs discussed e-cigarettes with their adolescent patients, compared to 60% for conventional cigarettes.<br>2. Nearly half (40%) reported that adolescents or parents never approached them about e-cigarettes.<br>3.The majority (79%) never recommended e-cigarettes as a cessation tool.<br>4. Providers has low confidence in advising about e-cigarettes (9% very confident) compared to conventional cigarettes (36% very confident); 32% had no confidence advising on e-cigarettes.		1.Cross-sectional design and a low response rate, leads to nonresponse bias<br>2.Not generalizable to the country or even state as there is only one response from the regional New South Wales state.<br>3.Small sample size and shorter study period<br>4.No statistical validation (e.g., principal components analysis or internal consistency analysis) was conducted, allowing for possible measurement biases.
Simoneau et al. (2021)	1. Clinicians (92%) indicated that e-cigarettes were equally or more dangerous to health than cigarettes	-	1. (57%) Counselled non-smoking adolescents about the dangers of EC at least annually<br>2. (34%) of providers never counselled their adolescent patients about the dangers of electronic cigarettes	1.Clinicians expressed a desire for an easy referral process and educational videos to help adolescents	1.Survey data was from 2016 did not asked type and content, knowledge on e-cigarette wasn't assessed properly.

Sundstrom et al. (2023)	Most providers (72.2%) had a satisfactory level of knowledge about e-cigarette devices, oral health effects, effects on brain development, gender prevalence in use, and association of e-cigarette use with socioeconomic status	Based on theory of planned behaviour following sections were measured<br><br>Behavioural belief<br><br>1. Majority 83% of participants were likely and extremely likely to ask their adolescent patient about e-cigarette use<br><br>2. 60% of participants believed documenting e-cigarette use in patient records was desirable or highly desirable when asking about e-cigarette use.<br>3. 53% believe asking adolescent patients about e-cigarette use would make them uncomfortable.<br><br>Normative beliefs<br><br>1. 22% of the sample reported that their dental hygienist friends ask adolescents about e-cigarette use.<br>2. About 39% indicated that most DHs do not think it ethically responsible to discuss e-cigarettes with patients<br>3. Only 37% of participants reported that their supervisors believe they should inquire about adolescent's e-cigarette use at every appointment.	-	-	1. Study use of a nonprobability sample may limit generalizability.<br><br>2. Participants' self-selection may impact external validity.<br><br>3. Nonresponse bias may affect the study, potentially skewing the results if certain groups are more likely to participate or not participate.<br><br>3. Participants may provide socially desirable responses.<br><br>4. The study's focus on the age of initiation in relation to a specific survey (Youth Risk Surveillance) may limit general knowledge assessment.<br><br>5. The study also faced limitations in adolescent e-cigarettes cessation tools

		Control beliefs<br><br>1. 63% of participants agreed they had time to ask adolescent patients about ENDS at every appointment.<br>2. Only 33% agreed or strongly agreed they had enough resources to ask adolescents about e-cigarettes at every appointment.<br><br>Behavioural intentions<br><br>About half, 42-53% of respondents agreed or strongly agreed on an intention to implement six behavioural strategies within the next six months, covering aspects such as inquiring about e-cigarette use, acquiring knowledge about the devices, ways to prevent use, factors influencing usage, and utilizing the 5 as interventions for e-cigarette use.			

Supplementary Table S2: Quality assessment checklist: CASP Tool

	Year	Purpose	Methodology	Research design	Recruitment sampling	Data collection	Ethics	Data analysis	Findings	Value of research	Overall score
Rajiv Singh et al.	2024	√	√	√	√	√	√	√	√	√	A
Sundstrom et al.	2023	√	√	√	√	√	√	√	√	*	B
Chin et al.	2022	√	√	√	√	√	√	√	√	√	A
Gorukanti et al.	2022	√	√	√	√	√	√	√	√	√	A
Metcalf et al	2022	√	√	√	√	√	√	√	√	√	A
Cano Rodriguez et al.	2021	√	√	√	√	√	*	√	√	*	B
McGee et al.	2021	√	√	√	√	√	√	√	√	√	A
Simoneau et al.	2021	√	√	√	√	√	√	√	√	√	A
Pepper et al.	2015	√	√	√	√	√	√	√	√	√	A
Pepper et al.	2014	√	√	√	√	√	√	√	√	√	A
Oliver et al.	2022	√	√	√	√	√	√	√	√	√	A
Peterson et al.	2018	√	√	√	√	√	√	√	√	√	A
Brown-Johnson et al.	2016	√	√	√	√	√	√	√	√	√	A
Gorzkowski et al.	2014	√	√	√	√	√	√	√	√	√	A

Legends: Key to ratings: ✓ = Detailed coverage of screening question; * = Screening question covered but not detailed; X= Screening question not addressed. Overall quality rating: A= Nil or few flaws, the study credibility, transferability, dependability, and confirmability is high; B= some flaws, unlikely to affect the credibility, transferability, dependability, and/or confirmability of the study; C = some flaws which may affect the credibility, transferability, and/or confirmability of the study; D = significant flaws which are very likely to affect the credibility, transferability, dependability and/or confirmability of the study(CASP 2023)**.**

S3: Search terms

CINAHL

S1(health care providers or professionals or doctor or nurse or physician or pediatrician or primary health care provider) AND (e-cigarettes or electronic cigarettes or vapor cigarettes or vapes or electronic nicotine delivery device or juul or vaping) AND (adolescents or teenagers or young adults or teen or youth or high school students or middle school students) AND (practices or strategies or approaches or knowledge or attitude or perception)

S2(healthcare personnel or healthcare worker or allied health professional or dentist) AND (e-cigarettes or vaping or electronic non nicotine delivery system or ENDS or ENNDS or electronic nicotine delivery system or juul) AND (adolescents or teenagers or young adults or teen or youth) AND (views or opinions or thoughts or experiences or attitudes or perceptions or beliefs)

MEDLINE:

S1 (health care providers or professionals or doctor or nurse or physician or pediatricianor primary health care provider) AND (e-cigarettes or electronic cigarettes or vapor cigarettes or vapes or electronic nicotine delivery device or juul or vaping) AND (adolescents or teenagers or young adults or teen or youth or high school students or middle school students) AND (practices or strategies or approaches or knowledge or attitude or perception)

S2(healthcare personnel or healthcare worker or allied health professional or dentist) AND (e-cigarettes or vaping or electronic non nicotine delivery system or ENDS or ENNDS or electronic nicotine delivery system or juul) AND (adolescents or teenagers or young adults or teen or youth) AND (views or opinions or thoughts or experiences or attitudes or perceptions or beliefs)

PubMed

#2(((health care providers or professionals or doctor or nurse or physician or pediatrician or primary health care provider) AND (e-cigarettes or electronic cigarettes or vapor cigarettes or vapes or electronic nicotine delivery device or juul or vaping)) AND (adolescents or teenagers or young adults or teen or youth or high school students or middle school students)) AND (practices or strategies or approaches or knowledge or attitude or perception)

((("delivery of health care"[MeSH Terms] OR ("delivery"[All Fields] AND "health"[All Fields] AND "care"[All Fields]) OR "delivery of health care"[All Fields] OR "healthcare"[All Fields] OR "healthcare s"[All Fields] OR "healthcares"[All Fields]) AND ("occupational groups"[MeSH Terms] OR ("occupational"[All Fields] AND "groups"[All Fields]) OR "occupational groups"[All Fields] OR "personnel"[All Fields] OR "personnel s"[All Fields] OR "personnels"[All Fields])) OR ("health personnel"[MeSH Terms] OR ("health"[All Fields] AND "personnel"[All Fields]) OR "health personnel"[All Fields] OR ("healthcare"[All Fields] AND "worker"[All Fields]) OR "healthcare worker"[All Fields]) OR ("allied health personnel"[MeSH Terms] OR ("allied"[All Fields] AND "health"[All Fields] AND "personnel"[All Fields]) OR "allied health personnel"[All Fields] OR ("allied"[All Fields] AND "health"[All Fields] AND "professional"[All Fields]) OR "allied health professional"[All Fields]) OR ("dentist s"[All Fields] OR "dentists"[MeSH Terms] OR "dentists"[All Fields] OR "dentist"[All Fields])) AND ("electronic nicotine delivery systems"[MeSH Terms] OR ("electronic"[All Fields] AND "nicotine"[All Fields] AND "delivery"[All Fields] AND "systems"[All Fields]) OR "electronic nicotine delivery systems"[All Fields] OR "e cigarettes"[All Fields] OR ("vaped"[All Fields] OR "vaping"[MeSH Terms] OR "vaping"[All Fields] OR "vapes"[All Fields]) OR (("electronical"[All Fields] OR "electronically"[All Fields] OR "electronics"[MeSH Terms] OR "electronics"[All Fields] OR "electronic"[All Fields]) AND "non"[All Fields] AND ("nicotine"[MeSH Terms] OR "nicotine"[All Fields] OR "nicotine s"[All Fields] OR "nicotines"[All Fields]) AND ("deliveries"[All Fields] OR "delivery, obstetric"[MeSH Terms] OR ("delivery"[All Fields] AND "obstetric"[All Fields]) OR "obstetric delivery"[All Fields] OR "delivery"[All Fields]) AND ("drug delivery systems"[MeSH Terms] OR ("drug"[All Fields] AND "delivery"[All Fields] AND "systems"[All Fields]) OR "drug delivery systems"[All Fields] OR "system"[All Fields] OR "system s"[All Fields] OR "systems"[All Fields])) OR "ENDS"[All Fields] OR "ENNDS"[All Fields] OR ("electronic nicotine delivery systems"[MeSH Terms] OR ("electronic"[All Fields] AND "nicotine"[All Fields] AND "delivery"[All Fields] AND "systems"[All Fields]) OR "electronic nicotine delivery systems"[All Fields] OR ("electronic"[All Fields] AND "nicotine"[All Fields] AND "delivery"[All Fields] AND "system"[All Fields]) OR "electronic nicotine delivery system"[All Fields]) OR "juul"[All Fields]) AND ("adolescences"[All Fields] OR "adolescency"[All Fields] OR "adolescent"[MeSH Terms] OR "adolescent"[All Fields] OR "adolescence"[All Fields] OR "adolescents"[All Fields] OR "adolescent s"[All Fields] OR ("adolescent"[MeSH Terms] OR "adolescent"[All Fields] OR "teenage"[All Fields] OR "teenager"[All Fields] OR "teenagers"[All Fields] OR "teenaged"[All Fields] OR "teenager s"[All Fields] OR "teenages"[All Fields]) OR ("young adult"[MeSH Terms] OR ("young"[All Fields] AND "adult"[All Fields]) OR "young adult"[All Fields] OR ("young"[All Fields] AND "adults"[All Fields]) OR "young adults"[All Fields]) OR ("adolescent"[MeSH Terms] OR "adolescent"[All Fields] OR "teen"[All Fields]) OR ("adolescent"[MeSH Terms] OR "adolescent"[All Fields] OR "youth"[All Fields] OR "youths"[All Fields] OR "youth s"[All Fields])) AND ("viewed"[All Fields] OR "viewing"[All

Fields] OR "viewings"[All Fields] OR "views"[All Fields] OR ("attitude"[MeSH Terms] OR "attitude"[All Fields] OR "opinion"[All Fields] OR "opinions"[All Fields] OR "opinion s"[All Fields] OR "opinionated"[All Fields]) OR ("thinking"[MeSH Terms] OR "thinking"[All Fields] OR "thought"[All Fields] OR "thoughts"[All Fields] OR "thought s"[All Fields] OR "thoughtful"[All Fields] OR "thoughtfulness"[All Fields]) OR ("experience"[All Fields] OR "experience s"[All Fields] OR "experiences"[All Fields]) OR ("attitude"[MeSH Terms] OR "attitude"[All Fields] OR "attitudes"[All Fields] OR "attitude s"[All Fields]) OR ("percept"[All Fields] OR "perceptibility"[All Fields] OR "perceptible"[All Fields] OR "perception"[MeSH Terms] OR "perception"[All Fields] OR "perceptions"[All Fields] OR "perceptional"[All Fields] OR "perceptive"[All Fields] OR "perceptiveness"[All Fields] OR "percepts"[All Fields]) OR ("belief s"[All Fields] OR "culture"[MeSH Terms] OR "culture"[All Fields] OR "belief"[All Fields] OR "beliefs"[All Fields]))

Translations

health care providers: "health personnel"[MeSH Terms] OR ("health"[All Fields] AND "personnel"[All Fields]) OR "health personnel"[All Fields] OR ("health"[All Fields] AND "care"[All Fields] AND "providers"[All Fields]) OR "health care providers"[All Fields]

professionals: "professional"[All Fields] OR "professional's"[All Fields] OR "professionalism"[MeSH Terms] OR "professionalism"[All Fields] OR "professionality"[All Fields] OR "professionalization"[All Fields] OR "professionalize"[All Fields] OR "professionalized"[All Fields] OR "professionalizing"[All Fields] OR "professionally"[All Fields] OR "professionals"[All Fields]

doctor: "doctor's"[All Fields] OR "doctoral"[All Fields] OR "doctorally"[All Fields] OR "doctorate"[All Fields] OR "doctorates"[All Fields] OR "doctoring"[All Fields] OR "physicians"[MeSH Terms] OR "physicians"[All Fields] OR "doctor"[All Fields] OR "doctors"[All Fields]

nurse: "nurse's"[All Fields] OR "nurses"[MeSH Terms] OR "nurses"[All Fields] OR "nurse"[All Fields] OR "nurses's"[All Fields]

physician: "physician's"[All Fields] OR "physicians"[MeSH Terms] OR "physicians"[All Fields] OR "physician"[All Fields] OR "physicians's"[All Fields]

pediatrician: "paediatrician's"[All Fields] OR "paediatricians"[All Fields] OR "pediatrician's"[All Fields] OR "pediatricians"[MeSH Terms] OR "pediatricians"[All Fields] OR "paediatrician"[All Fields] OR "pediatrician"[All Fields]

primary health care: "primary health care"[MeSH Terms] OR ("primary"[All Fields] AND "health"[All Fields] AND "care"[All Fields]) OR "primary health care"[All Fields]

provider: "provide"[All Fields] OR "provided"[All Fields] OR "provider"[All Fields] OR "provider's"[All Fields] OR "providers"[All Fields] OR "provides"[All Fields] OR "providing"[All Fields]

e-cigarettes: "electronic nicotine delivery systems"[MeSH Terms] OR ("electronic"[All Fields] AND "nicotine"[All Fields] AND "delivery"[All Fields] AND "systems"[All Fields]) OR "electronic nicotine delivery systems"[All Fields] OR "e cigarettes"[All Fields]

electronic cigarettes: "electronic nicotine delivery systems"[MeSH Terms] OR ("electronic"[All Fields] AND "nicotine"[All Fields] AND "delivery"[All Fields] AND "systems"[All Fields]) OR "electronic nicotine delivery systems"[All Fields] OR ("electronic"[All Fields] AND "cigarettes"[All Fields]) OR "electronic cigarettes"[All Fields]
vapor: "nebulizers and vaporizers"[MeSH Terms] OR ("nebulizers"[All Fields] AND "vaporizers"[All Fields]) OR "nebulizers and vaporizers"[All Fields] OR "vaporizers"[All Fields] OR "vapor"[All Fields] OR "vaporisation"[All Fields] OR "volatilization"[MeSH Terms] OR "volatilization"[All Fields] OR "vaporization"[All Fields] OR "laser therapy"[MeSH Terms] OR ("laser"[All Fields] AND "therapy"[All Fields]) OR "laser therapy"[All Fields] OR "vaporise"[All Fields] OR "vaporised"[All Fields] OR "vaporiser"[All Fields] OR "vaporizer"[All Fields] OR "vaporisers"[All Fields] OR "vaporises"[All Fields] OR "vaporizations"[All Fields] OR "vaporize"[All Fields] OR "vaporized"[All Fields] OR "vaporizes"[All Fields] OR "vaporizing"[All Fields] OR "vaporous"[All Fields] OR "vapors"[All Fields] OR "vapour"[All Fields] OR "vapourization"[All Fields] OR "vapourized"[All Fields] OR "vapours"[All Fields]
cigarettes: "cigarett"[All Fields] OR "cigarette's"[All Fields] OR "cigaretts"[All Fields] OR "tobacco products"[MeSH Terms] OR ("tobacco"[All Fields] AND "products"[All Fields]) OR "tobacco products"[All Fields] OR "cigarette"[All Fields] OR "cigarettes"[All Fields]
vapes: "vaped"[All Fields] OR "vaping"[MeSH Terms] OR "vaping"[All Fields] OR "vapes"[All Fields]
electronic: "electronical"[All Fields] OR "electronically"[All Fields] OR "electronics"[MeSH Terms] OR "electronics"[All Fields] OR "electronic"[All Fields]
nicotine delivery device: "tobacco use cessation devices"[MeSH Terms] OR ("tobacco"[All Fields] AND "cessation"[All Fields] AND "devices"[All Fields]) OR "tobacco use cessation devices"[All Fields] OR ("nicotine"[All Fields] AND "delivery"[All Fields] AND "device"[All Fields]) OR "nicotine delivery device"[All Fields]
vaping: "vaped"[All Fields] OR "vaping"[MeSH Terms] OR "vaping"[All Fields] OR "vapes"[All Fields]
adolescents: "adolescences"[All Fields] OR "adolescency"[All Fields] OR "adolescent"[MeSH Terms] OR "adolescent"[All Fields] OR "adolescence"[All Fields] OR "adolescents"[All Fields] OR "adolescent's"[All Fields]
teenagers: "adolescent"[MeSH Terms] OR "adolescent"[All Fields] OR "teenage"[All Fields] OR "teenager"[All Fields] OR "teenagers"[All Fields] OR "teenaged"[All Fields] OR "teenager's"[All Fields] OR "teenages"[All Fields]
young adults: "young adult"[MeSH Terms] OR ("young"[All Fields] AND "adult"[All Fields]) OR "young adult"[All Fields] OR ("young"[All Fields] AND "adults"[All Fields]) OR "young adults"[All Fields]
teen: "adolescent"[MeSH Terms] OR "adolescent"[All Fields] OR "teen"[All Fields]
youth: "adolescent"[MeSH Terms] OR "adolescent"[All Fields] OR "youth"[All Fields] OR "youths"[All Fields] OR "youth's"[All Fields]
school: "educational status"[MeSH Terms] OR ("educational"[All Fields] AND "status"[All Fields]) OR "educational status"[All Fields] OR "schooling"[All Fields] OR "education"[MeSH Terms] OR "education"[All Fields] OR "school's"[All Fields] OR "schooled"[All Fields] OR "schools"[MeSH Terms] OR "schools"[All Fields] OR "school"[All Fields]

students: "student's"[All Fields] OR "students"[MeSH Terms] OR "students"[All Fields] OR "student"[All Fields] OR "students's"[All Fields]
middle: "middle"[All Fields] OR "middles"[All Fields]
school: "educational status"[MeSH Terms] OR ("educational"[All Fields] AND "status"[All Fields]) OR "educational status"[All Fields] OR "schooling"[All Fields] OR "education"[MeSH Terms] OR "education"[All Fields] OR "school's"[All Fields] OR "schooled"[All Fields] OR "schools"[MeSH Terms] OR "schools"[All Fields] OR "school"[All Fields]
students: "student's"[All Fields] OR "students"[MeSH Terms] OR "students"[All Fields] OR "student"[All Fields] OR "students's"[All Fields]
practices: "practicability"[All Fields] OR "practicable"[All Fields] OR "practical"[All Fields] OR "practicalities"[All Fields] OR "practicality"[All Fields] OR "practically"[All Fields] OR "practicals"[All Fields] OR "practice"[All Fields] OR "practice's"[All Fields] OR "practiced"[All Fields] OR "practices"[All Fields] OR "practicing"[All Fields]
strategies: "strategie"[All Fields] OR "strategies"[All Fields] OR "strategy"[All Fields] OR "strategy's"[All Fields]
approaches: "approach"[All Fields] OR "approach's"[All Fields] OR "approachability"[All Fields] OR "approachable"[All Fields] OR "approache"[All Fields] OR "approached"[All Fields] OR "approaches"[All Fields] OR "approaching"[All Fields] OR "approachs"[All Fields]
knowledge: "knowledge"[MeSH Terms] OR "knowledge"[All Fields] OR "knowledge's"[All Fields] OR "knowledgeability"[All Fields] OR "knowledgeable"[All Fields] OR "knowledgeably"[All Fields] OR "knowledges"[All Fields]
attitude: "attitude"[MeSH Terms] OR "attitude"[All Fields] OR "attitudes"[All Fields] OR "attitude's"[All Fields]
perception: "percept"[All Fields] OR "perceptibility"[All Fields] OR "perceptible"[All Fields] OR "perception"[MeSH Terms] OR "perception"[All Fields] OR "perceptions"[All Fields] OR "perceptional"[All Fields] OR "perceptive"[All Fields] OR "perceptiveness"[All Fields] OR "percepts"[All Fields]

PsycInfo

S1(health care providers or professionals or doctor or nurse or physician or pediatrician or primary health care provider) AND (e-cigarettes or electronic cigarettes or vapor cigarettes or vapes or electronic nicotine delivery device or juul or vaping) AND (adolescents or teenagers or young adults or teen or youth or high school students or middle school students) AND (practices or strategies or approaches or knowledge or attitude or perception)

S2(healthcare personnel or healthcare worker or allied health professional or dentist) AND (e-cigarettes or vaping or electronic non nicotine delivery system or ENDS or ENNDS or electronic nicotine delivery system or juul) AND (adolescents or teenagers or young adults or teen or youth) AND (views or opinions or thoughts or experiences or attitudes or perceptions or beliefs)

Scopus

#1
#health AND care AND providers OR professionals OR doctor OR nurse OR physician OR pediatrician OR primary AND health AND care A
ND provider AND e-
cigarettes OR electronic AND cigarettes OR vapor AND cigarettes OR vapes OR electronic AND nicotine AND delivery AND device OR juul O
R vapingadolescents OR teenagers OR young AND adults OR teen OR youth OR high AND school AND students OR middle AND school AN
D students AND practices OR strategies OR approaches OR knowledge OR attitude OR perception

#2
healthcare AND personnel OR healthcare AND worker OR allied AND health AND professional OR dentist AND ecigarettes OR vaping OR el
ectronic AND non AND nicotine AND delivery AND system OR ends OR ennds OR electronic AND nicotine AND delivery AND system OR j
uul AND adolescents OR teenagers OR young AND adults OR teen OR youth AND views OR opinions OR thoughts OR experiences OR attitu
des OR perceptions OR beliefs

(health AND care AND providers OR professionals OR doctor OR nurse OR physician OR pediatrician OR primary AND health AND care AND provider AND ecigarettes OR electronic AND cigarettes OR vapor AND cigarettes OR vapes OR electronic AND nicotine AND delivery AND device OR juul OR vapingadolescents OR teenagers OR young AND adults OR teen OR youth OR high AND school AND students OR middle AND school AND students AND practices OR strategies OR approaches OR knowledge OR attitude OR perception) OR (healthcare AND personnel OR healthcare AND worker OR allied AND health AND professional OR dentist AND e-cigarettes OR vaping OR electronic AND non AND nicotine AND delivery AND system OR ends OR ennds OR electronic AND nicotine AND delivery AND system OR juul AND adolescents OR teenagers OR young AND adults OR teen OR youth AND views OR opinions OR thoughts OR experiences OR attitudes OR perceptions OR beliefs)
